# Genetic linkage of hyperglycemia and dyslipidemia in an intercross between BALB/cJ and SM/J *Apoe*-deficient mouse strains

**DOI:** 10.1186/s12863-015-0292-y

**Published:** 2015-11-10

**Authors:** Qian Wang, Andrew T. Grainger, Ani Manichaikul, Emily Farber, Suna Onengut-Gumuscu, Weibin Shi

**Affiliations:** Department of Radiology & Medical Imaging, University of Virginia, Snyder Bldg Rm 266, 480 Ray C. Hunt Dr., P.O. Box 801339, Fontaine Research Park, Charlottesville, VA 22908 USA; University of Virginia, Snyder Bldg Rm 266, 480 Ray C. Hunt Dr., P.O. Box 801339, Fontaine Research Park, Charlottesville, VA 22908 USA; Department of Biochemistry & Molecular Genetics, University of Virginia, Charlottesville, VA USA; University of Virginia, Charlottesville, VA USA; Center for Public Health and Genomics, University of Virginia, Charlottesville, VA USA

**Keywords:** Dyslipidemia, Hyperglycemia, Type 2 diabetes, Quantitative trait locus, Genetic linkage

## Abstract

**Background:**

Individuals with dyslipidemia often develop type 2 diabetes, and diabetic patients often have dyslipidemia. It remains to be determined whether there are genetic connections between the 2 disorders.

**Methods:**

A female F_2_ cohort, generated from BALB/cJ (BALB) and SM/J (SM) *Apoe*-deficient (*Apoe*^−/−^) strains, was started on a Western diet at 6 weeks of age and maintained on the diet for 12 weeks. Fasting plasma glucose and lipid levels were measured before and after 12 weeks of Western diet. 144 genetic markers across the entire genome were used for quantitative trait locus (QTL) analysis.

**Results:**

One significant QTL on chromosome 9, named *Bglu17* [26.4 cM, logarithm of odds ratio (LOD): 5.4], and 3 suggestive QTLs were identified for fasting glucose levels. The suggestive QTL near the proximal end of chromosome 9 (2.4 cM, LOD: 3.12) was replicated at both time points and named *Bglu16. Bglu17* coincided with a significant QTL for HDL (high-density lipoprotein) and a suggestive QTL for non-HDL cholesterol levels. Plasma glucose levels were inversely correlated with HDL but positively correlated with non-HDL cholesterol levels in F_2_ mice on either chow or Western diet. A significant correlation between fasting glucose and triglyceride levels was also observed on the Western diet. Haplotype analysis revealed that “lipid genes” *Sik3, Apoa1,* and *Apoc3* were probable candidates for *Bglu17.*

**Conclusions:**

We have identified multiple QTLs for fasting glucose and lipid levels. The colocalization of QTLs for both phenotypes and the sharing of potential candidate genes demonstrate genetic connections between dyslipidemia and type 2 diabetes.

**Electronic supplementary material:**

The online version of this article (doi:10.1186/s12863-015-0292-y) contains supplementary material, which is available to authorized users.

## Background

Individuals with dyslipidemia have an increased risk of developing type 2 diabetes (T2D), and diabetic patients often have dyslipidemia, which includes elevations in plasma triglyceride and low-density lipoprotein (LDL) cholesterol levels and reductions in high-density lipoprotein (HDL) cholesterol levels [1]. Part of the increased diabetic risk associated with dyslipidemia is due to genetic variations that influence both lipoprotein homeostasis and the development of T2D. Indeed, a few rare gene mutations result in both dyslipidemia and T2D, which include *ABCA1* [2]*, LIPE* [3], *LPL* [4], and *LRP6* [5]. Genome-wide association studies (GWAS) have identified >150 loci associated to variation in plasma lipids [6, 7] and >70 loci associated with T2D, fasting plasma glucose, glycated hemoglobin (HbA1c), or insulin resistance [8–10]. Nearly a dozen of the loci detected are associated with both lipid and T2D-related traits at the genome-wide significance level, including *GCKR*, *FADS1*, *IRS1*, *KLF14*, and *HFE* (http://www.genome.gov/GWAStudies/). Surprisingly, half of them have shown opposite allelic effect on dyslipidemia and glucose levels [11], and this is in contrary to the positive correlations observed at the clinical level. Furthermore, it is challenging to establish causality between genetic variants and complex traits in humans due to small gene effects, complex genetic structure, and environmental influences.

A complementary approach to finding genetic components in human disease is to use animal models. Apolipoprotein E-deficient (*Apoe*^−/−^) mice are a commonly used mouse model of dyslipidemia, with elevations in non-HDL cholesterol levels and reductions in HDL levels, even when fed a low fat chow diet [12, 13]. High fat diet feeding aggravates dyslipidemia. Moreover, these mice develop all phases of atherosclerotic lesions seen in humans [14] and are extensively used for atherosclerosis research [15–18]. We have found that *Apoe*^−/−^ mice with certain genetic backgrounds develop significant hyperglycemia and T2D when fed a Western-type diet but become resistant with some other genetic backgrounds [16, 19, 20]. BALB/cJ (BALB) and SM/J (SM) *Apoe*^−/−^ mice exhibit differences in dyslipidemia and T2D-related phenotypes [16]. The objective of the present study was to explore potential genetic connections between dyslipidemia and T2D through quantitative trait locus (QTL) analysis of a female cohort derived from an intercross between BALB-*Apoe*^−/−^ and SM-*Apoe*^−/−^ mice.

## Methods

### Ethics statement

All procedures were in accordance with current National Institutes of Health guidelines (https://grants.nih.gov/grants/olaw/Guide-for-the-Care-and-use-of-laboratory-animals.pdf) and approved by the institutional Animal Care and Use Committee (protocol #: 3109). Blood was drawn from the retro-orbital plexus of overnight fasted mice with the animals under isoflurane anesthesia.

### Animals, experimental design and procedures

BALB and SM *Apoe*^−/−^ mice were created using the classic congenic breeding strategy, as described [16]. BALB-*Apoe*^−/−^ mice were crossed with SM-*Apoe*^−/−^ mice to generate F_1_s, which were intercrossed by brother-sister mating to generate a female F_2_ cohort. Mice were weaned at 3 weeks of age onto a rodent chow diet. At 6 weeks of age, female F_2_ mice were started on a Western diet containing 21 % fat, 34.1 % sucrose, 0.15 % cholesterol, and 19.5 % casein *by weight* (Harlan Laboratories, TD 88137) and maintained on the diet for 12 weeks. Mice were bled twice: once before initiation of the Western diet and once at the end of the 12-week feeding period. Overnight fasted mice were bled into tubes containing 8 μL of 0.5 mol/L ethylenediaminetetraacetic acid. Plasma was prepared and stored at −80 °C before use.

### Housing and husbandry

Breeding pairs were housed in a cage of 1 adult male and 2 females, and litters were weaned at 3 weeks of age onto a rodent chow diet in a cage of 5 or less. At 6 weeks of age, F_2_ mice were switched onto the Western diet and maintained on the diet for 12 weeks. All mice were housed under a 12-h light/dark cycle at an ambient temperature of 23 °C and allowed free access to water and drinking food. Mice were fasted overnight before blood samples were collected.

### Measurements of plasma glucose and lipid levels

Plasma glucose was measured with a Sigma glucose (HK) assay kit, as reported with modification to a longer incubation time [21]. Briefly, 6 μl of plasma samples were incubated with 150 μl of assay reagent in a 96-well plate for 30 min at 30 °C. The absorbance at 340 nm was read on a Molecular Devices (Menlo Park, CA) plate reader. The measurements of total cholesterol, HDL cholesterol, and triglyceride were performed as reported previously [13]. Non-HDL cholesterol was calculated as the difference between total and HDL cholesterol.

### Genotyping

Genomic DNA was isolated from the tails of mice by using the phenol/chloroform extraction and ethanol precipitation method. The Illumina LD linkage panel consisting of 377 SNP loci was used to genotype the F_2_ cohort. Microsatellite markers were typed for chromosome 8 where SNP markers were uninformative in distinguishing the parental origin of alleles. DNA samples from the two parental strains and their F_1_s served as controls. Uninformative SNPs were excluded from QTL analysis. SNP markers were also filtered based on the expected pattern in the control samples, and F_2_ mice were filtered based on 95 % call rates in genotype calls. After filtration, 228 F_2_s and 144 markers were included in genome-wide QTL analysis.

### Statistical analysis

QTL analysis was performed using J/qtl and Map Manager QTX software as previously reported [19, 22, 23]. One thousand permutations of trait values were run to define the genome-wide LOD (logarithm of odds) score threshold needed for significant or suggestive linkage of each trait. Loci that exceeded the 95th percentile of the permutation distribution were defined as significant (*P* < 0.05) and those exceeding the 37th percentile were suggestive (*P* < 0.63).

### Prioritization of positional candidate genes

The Sanger SNP database (http://www.sanger.ac.uk/sanger/Mouse_SnpViewer/rel-1410) was used to prioritize candidate genes for overlapping QTLs affecting plasma glucose and HDL cholesterol levels on chromosome (Chr) 9, which were mapped in two or more crosses derived from different parental strains for either phenotype. We converted the original mapping positions in cM for the confidence interval to physical positions in Mb and then examined SNPs within the confidence interval. Probable candidate genes were defined as those with one or more SNPs in coding or upstream promoter regions that were shared by the parental strains carrying the “high” allele but were different from the parental strains carrying the “low” allele at a QTL, as previously reported [24].

## Results

### Trait value distributions

**Fig. 1:**
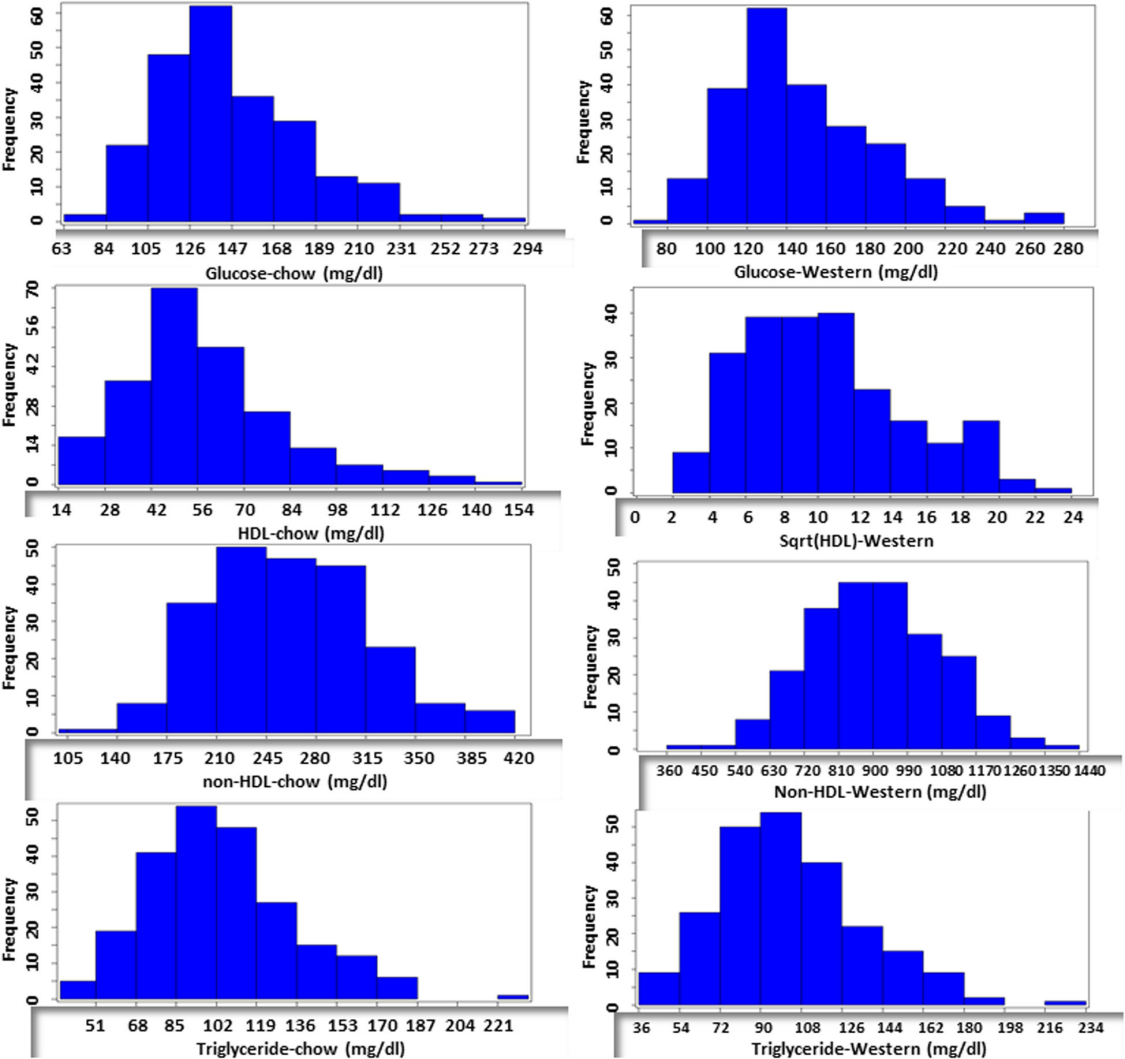
The distributions of trait values for fasting plasma glucose, HDL, non-HDL cholesterol and triglyceride of 228 female F_2_ mice derived from an intercross between BALB-*Apoe* ^−/−^ and SM-*Apoe* ^−/−^ mice. Fasting blood was collected once before initiation of the Western diet (left panel) and once after 12 weeks on the Western diet (right panel). Graphs were created using a plotting function of J/qtl software

Fasting plasma glucose and lipid levels of F_2_ mice were measured before and after 12-weeks of Western diet. Values of fasting plasma glucose, non-HDL cholesterol and triglyceride levels of F_2_ mice on both chow and Western diets and of HDL cholesterol level on the chow diet were normally or approximately normally distributed (Fig. 1). Values of square root-transformed HDL cholesterol levels on the Western diet showed a normal distribution. These data were then analyzed to search for QTLs affecting the traits. Loci with a genome-wide suggestive or significant *P* value are presented in Table 1.

**Table 1:** Significant and suggestive QTLs for plasma glucose and lipid levels in female F_2_ mice derived from BALB-*Apoe* ^−/−^ and SM-*Apoe* ^−/−^ mice

Locus	Chr	Trait	LOD^a^	*p*-value^b^	Peak (cM)	95 % CI^c^	High allele	Mode of inheritence^d^
*Bglu16*	9	Glucose-C	2.214	0.549	2.37	0.37–30.37	B	Additive
*Bglu13*	5	Glucose-W	2.1.8	<0.63	67.4	45.4–80.03	S	Recessive
-	5	Glucose-W	3.198	0.097	101.24	29.40–101.24	S	Additive
*Bglu16*	9	Glucose-W	3.12	<0.63	2.37	0–10.37	B	Additive
***Bglu17***	9	Glucose-W	**5.425**	**0.001**	26.37	16.37–40.37	B	Additive
***Hdlq5***	1	HDL-C	**8.64**	**0.000**	93.52	87.52–97.02	S	Additive
*Hdlcl1*	7	HDL-C	2.668	0.321	61.33	35.57–89.57	S	Dominant
***Hdlq17***	9	HDL-C	**4.614**	**0.014**	30.37	16.37–32.37	S	Additive
***Hdlq26***	10	HDL-C	2.181	0.591	61.22	25.03–61.22	S	Dominant
***Hdlq5***	1	HDL-W	**13.944**	**0.000**	87.52	83.52–93.52	S	Additive
***Hdlcl1***	7	HDL-W	**3.658**	**0.034**	85.57	77.57–89.67	S	Additive
***Hdlq17***	9	HDL-W	**10.625**	**0.000**	30.42	24.37–30.53	S	Additive
*Chol7*	1	non-HDL-C	2.093	0.626	66.95	9.52–74.56	B	Recessive
*Nhdlq15*	2	non-HDL-C	2.56	0.321	23.86	8.73–38.73	B	Additive
*Hdlq34*	5	non-HDL-C	2.106	0.614	19.4	19.4–30.5	S	Additive
*Pnhdlc1*	6	non-HDL-C	2.489	0.362	57.53	1.53–77.53	B	Recessive
*Nhdlq1*	8	non-HDL-C	2.221	0.537	44.14	10.14–60.14	B	Additive
*Nhdlq12*	12	non-HDL-C	2.73	0.245	39.41	15.41–59.41	B	Additive
***Nhdlq15***	**2**	**non-HDL-W**	**4.79**	**0.002**	31.80	22.73–40.73	B	Dominant
*Nhdlq11*	9	non-HDL-W	2.136	0.585	32.37	0.37–75.33	B	Additive
*-*	11	non-HDL-W	2.332	0.436	1.99	1.99–17.99	B	Dominant
***Nhdlq16***	**16**	**non-HDL-W**	**3.99**	**0.011**	46.66	35.43–46.66	S	Dominant
*Tgq11*	2	Triglyceride-C	2.952	0.169	26.73	12.73–60.83	B	Additive
*-*	5	Triglyceride-C	2.759	0.234	80.03	73.40–93.40	S	Heterosis
*Trglyd*	1	Triglyceride-W	3.291	0.091	97.02	79.24–97.02	S	Additive

^a^LOD scores were obtained from genome-wide QTL analysis using J/qtl software. The significant LOD scores were highlighted in bold. The suggestive and significant LOD score thresholds were determined by 1,000 permutation tests for each trait. Suggestive and significant LOD scores were 2.116 and 3.429, respectively, for glucose on the chow diet; 2.056 and 3.569 for glucose on the Western diet; 2.127 and 3.725 for HDL cholesterol, 2.09 and 3.662 for non-HDL cholesterol, and 2.102 and 3.522 for triglyceride on the chow diet; 2.10 and 3.486 for HDL, 2.123 and 3.628 for non-HDL, and 2.123 and 3.628 for triglyceride on the Western diet

^b^The *p*-values reported represent the level of genome-wide significance

^c^95 % Confidence interval in cM defined by a whole genome QTL scan

^d^Mode of inheritance was defined according to allelic effect at the nearest marker of a QTL

### Fasting glucose levels

**Fig. 2:**
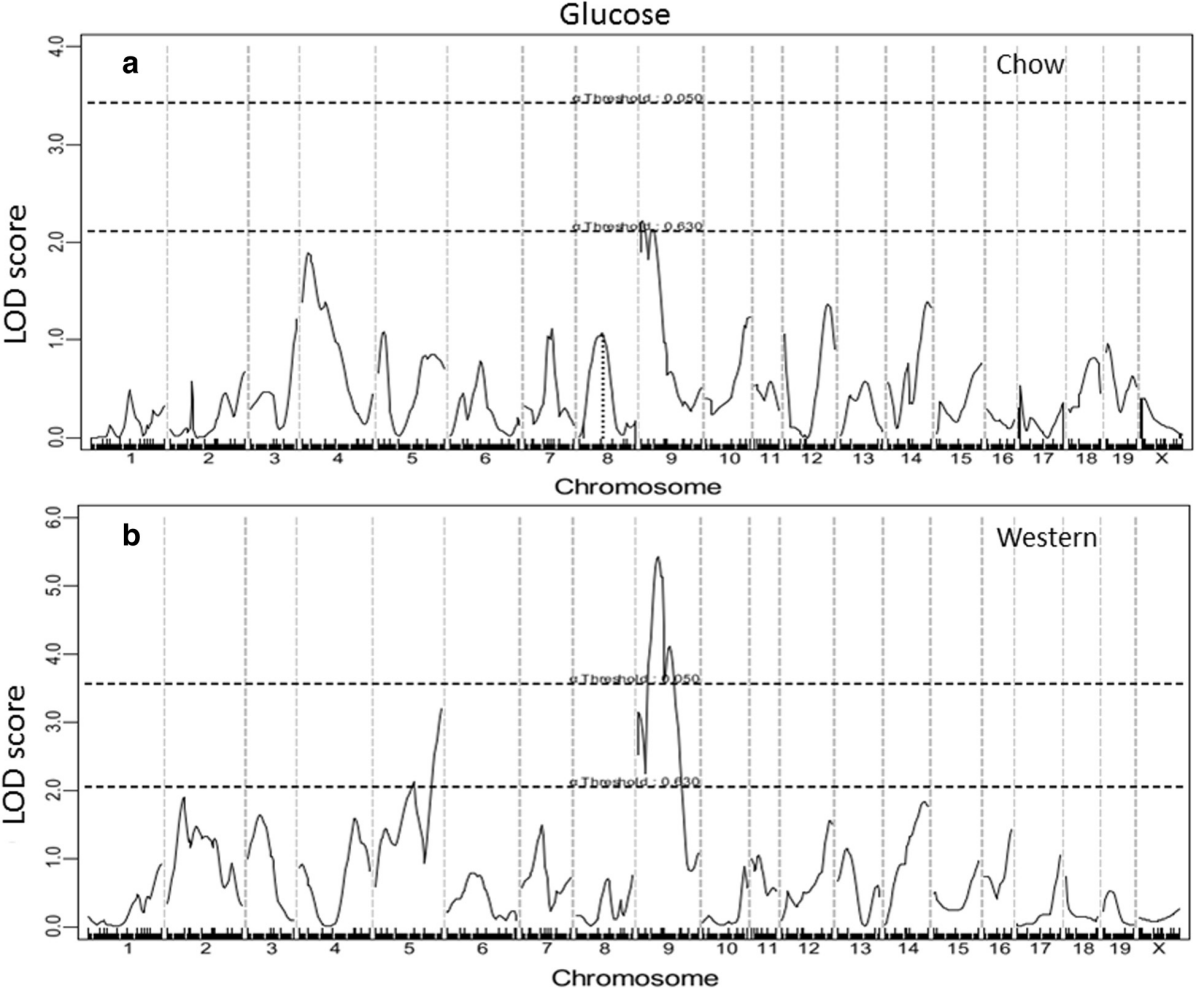
Genome-wide scans to search for main effect loci influencing fasting plasma glucose levels of female F_2_ mice when fed a chow (**a**) or Western diet (**b**). Chromosomes 1 through X are represented numerically on the X-axis. The Y-axis represents the LOD score. Two horizontal dashed lines denote genome-wide empirical thresholds for suggestive (*P* = 0.63) and significant (*P* = 0.05) linkage

A genome-wide scan for main effect QTL revealed a suggestive QTL near the proximal end of Chr9 for fasting glucose when mice were fed the chow diet (2.37 cM, LOD: 2.21) (Fig. 2 and Table 1). As this QTL was replicated on the Western diet, it was named *Bglu16.* For fasting glucose levels on the Western diet, a significant QTL on Chr9 and 3 suggestive QTLs, including *Bglu16* on Chr9, were identified. The significant QTL on Chr9 peaked at 26.37 cM and had a LOD score of 5.425. It was named *Bglu17.* The suggestive QTL near the middle portion of Chr5 (67.4 cM, LOD 2.18) replicated *Bglu13*, initially mapped in a B6 x BALB *Apoe*^−/−^ intercross [21]. The suggestive QTL on distal Chr5 (101.24 cM, LOD 3.198) was novel. The BALB allele conferred an increased glucose level for both of the Chr9 QTLs while the SM allele conferred increased glucose levels for the 2 Chr5 QTLs (Table 2).

**Table 2:** Allelic effects in different QTLs on plasma glucose and lipids of female F_2_ mice derived from BALB and SM *Apoe* ^−/−^ mice

Locus name	Chr	Trait	LOD	Peak (cM)	Closest marker	BB	SS	SB
*Bglu16*	9	Glucose-C	2.214	2.37	rs13480073	109.0 ± 28.7 (*n* = 44)	93.9 ± 22.9 (*n* = 43)	97.4 ± 22.6 (*n* = 141)
*Bglu13*	5	Glucose-W	2.1.8	67.4	rs3726547	144.4 ± 30.6 (*n* = 43)	153.5 ± 40.4 (*n* = 88)	142.9 ± 35.3 (*n* = 97)
-	5	Glucose-W	3.198	101.24	rs13478578	132.7 ± 31.3 (*n* = 51)	158.8 ± 41.8 (*n* = 63)	147.5 ± 34.0 (*n* = 113)
*Bglu16*	9	Glucose-W	3.12	2.37	rs13480073	165.3 ± 40.9 (*n* = 54)	138.3 ± 30.0 (*n* = 43)	144.4 ± 35.7 (*n* = 141)
***Bglu17***	9	Glucose-W	**5.425**	26.37	CEL.9_49183636	168.0 ± 39.8 (*n* = 42)	134.7 ± 26.5 (*n* = 62)	146.1 ± 37.1 (*n* = 124)
***Hdlq5***	1	HDL-C	**8.64**	93.52	rs13476259	49.5 ± 20.9 (*n* = 60)	73.2 ± 26.5 (*n* = 62)	55.1 ± 19.4 (*n* = 106)
*Hdlcl1*	7	HDL-C	2.668	61.33	rs3724711	49.0 ± 20.0 (*n* = 50)	58.3 ± 21.0 (*n* = 63)	62.9 ± 25.5 (*n* = 115)
***Hdlq17***	9	HDL-C	**4.614**	30.37	CEL.9_49183636	49.5 ± 15.9 (*n* = 42)	69.4 ± 26.8 (*n* = 62)	56.2 ± 22.5 (*n* = 124)
*Hdlq26*	10	HDL-C	2.181	61.22	rs3688351	50.7 ± 19.2 (*n* = 60)	59.4 ± 21.9 (*n* = 53)	62.4 ± 25.8 (*n* = 114)
***Hdlq5***	1	sqrtHDL-W	**13.944**	87.52	rs3685643	66.6 ± 50.2 (*n* = 57)	201.1 ± 118.8 (*n* = 62)	117.0 ± 97.1 (*n* = 109)
***Hdlcl1***	7	sqrtHDL-W	**3.658**	85.57	rs6216320	95.5 ± 87.6 (*n* = 63)	173.3 ± 126.7 (*n* = 55)	122.4 ± 98.4 (*n* = 110)
***Hdlq17***	9	sqrtHDL-W	**10.625**	30.42	CEL.9_49183636	57.6 ± 49.3 (*n* = 42)	183.3 ± 115.0 (*n* = 62)	122.8 ± 101.7 (*n* = 124)
*Chol7*	1	non-HDL-C	2.093	66.95	rs6354736	279.5 ± 62.8 (*n* = 56)	257.8 ± 56.9 (*n* = 57)	251.2 ± 52.1 (*n* = 114)
*Nhdlq15*	2	non-HDL-C	2.56	23.86	mCV23209429	273.9 ± 56.1 (*n* = 55)	238.0 ± 47.0 (*n* = 53)	262.7 ± 59.0 (*n* = 120)
*Hdlq34*	5	non-HDL-C	2.106	19.4	rs3658401	244.5 ± 54.7 (*n* = 63)	276.0 ± 53.2 (*n* = 61)	259.3 ± 58.3 (*n* = 104)
*Pnhdlc1*	6	non-HDL-C	2.489	57.53	rs13478909	279.6 ± 51.3 (*n* = 51)	252.0 ± 65.2 (*n* = 57)	254.8 ± 53.5 (*n* = 120)
*Nhdlq1*	8	non-HDL-C	2.221	44.14	D8Mit50	275.0 ± 54.5 (*n* = 60)	242.4 ± 57.4 (*n* = 57)	262.9 ± 55.6 (*n* = 96)
*Nhdlq12*	12	non-HDL-C	2.73	39.41	rs6195664	278.6 ± 52.3 (*n* = 62)	243.8 ± 57.8 (*n* = 59)	257.4 ± 56.5 (*n* = 107)
***Nhdlq15***	**2**	non-HDL-W	**4.79**	31.8	rs13476507	954.1 ± 156.0 (*n* = 56)	806.9 ± 158.2 (*n* = 47)	915.6 ± 166.1 (*n* = 125)
*Nhdlq11*	9	non-HDL-W	2.136	32.37	rs3709825	958.4 ± 211.4 (*n* = 42)	856.8 ± 165.3 (*n* = 62)	906.6 ± 149.5 (*n* = 124)
*-*	11	non-HDL-W	2.332	1.99	rs4222040	927.3 ± 149.8 (*n* = 67)	849.0 ± 165.1 (*n* = 69)	917.0 ± 170.5 (*n* = 85)
***Nhdlq16***	**16**	non-HDL-W	**3.99**	46.66	rs3721202	820.2 ± 152.7 (*n* = 56)	931.9 ± 146.4 (*n* = 52)	928.4 ± 174.6 (*n* = 120)
*Tgq11*	2	Triglyceride-C	2.952	26.73	mCV23209429	123.7 ± 35.7 (*n* = 55)	101.9 ± 34.6 (*n* = 53)	107.3 ± 31.8 (*n* = 120)
*-*	5	Triglyceride-C	2.759	80.03	gnf05.120.578	110.2 ± 33.2 (*n* = 43)	119.3 ± 35.8 (*n* = 88)	101.6 ± 31.3 (*n* = 97)
*Trglyd*	1	Triglyceride-W	3.291	97.02	rs13476259	94.0 ± 28.6 (*n* = 59)	115.3 ± 33.0 (*n* = 62)	100.7 ± 30.8 (*n* = 106)
*Bglu16*	9	Glucose-C	2.214	2.37	rs13480073	109.0 ± 28.7 (*n* = 44)	93.9 ± 22.9 (*n* = 43)	97.4 ± 22.6 (*n* = 141)
*Bglu13*	5	Glucose-W	2.1.8	67.4	rs3726547	144.4 ± 30.6 (*n* = 43)	153.5 ± 40.4 (*n* = 88)	142.9 ± 35.3 (*n* = 97)
-	5	Glucose-W	3.198	101.24	rs13478578	132.7 ± 31.3 (*n* = 51)	158.8 ± 41.8 (*n* = 63)	147.5 ± 34.0 (*n* = 113)
*Bglu16*	9	Glucose-W	3.12	2.37	rs13480073	165.3 ± 40.9 (*n* = 54)	138.3 ± 30.0 (*n* = 43)	144.4 ± 35.7 (*n* = 141)
***Bglu17***	9	Glucose-W	**5.425**	26.37	CEL.9_49183636	168.0 ± 39.8 (*n* = 42)	134.7 ± 26.5 (*n* = 62)	146.1 ± 37.1 (*n* = 124)
***Hdlq5***	1	HDL-C	**8.64**	93.52	rs13476259	49.5 ± 20.9 (*n* = 60)	73.2 ± 26.5 (*n* = 62)	55.1 ± 19.4 (*n* = 106)
*Hdlcl1*	7	HDL-C	2.668	61.33	rs3724711	49.0 ± 20.0 (*n* = 50)	58.3 ± 21.0 (*n* = 63)	62.9 ± 25.5 (*n* = 115)
***Hdlq17***	9	HDL-C	**4.614**	30.37	CEL.9_49183636	49.5 ± 15.9 (*n* = 42)	69.4 ± 26.8 (*n* = 62)	56.2 ± 22.5 (*n* = 124)
*Hdlq26*	10	HDL-C	2.181	61.22	rs3688351	50.7 ± 19.2 (*n* = 60)	59.4 ± 21.9 (*n* = 53)	62.4 ± 25.8 (*n* = 114)
***Hdlq5***	1	sqrtHDL-W	**13.944**	87.52	r[55]s3685643	66.6 ± 50.2 (*n* = 57)	201.1 ± 118.8 (*n* = 62)	117.0 ± 97.1 (*n* = 109)
***Hdlcl1***	7	sqrtHDL-W	**3.658**	85.57	rs6216320	95.5 ± 87.6 (*n* = 63)	173.3 ± 126.7 (*n* = 55)	122.4 ± 98.4 (*n* = 110)
***Hdlq17***	9	sqrtHDL-W	**10.625**	30.42	CEL.9_49183636	57.6 ± 49.3 (*n* = 42)	183.3 ± 115.0 (*n* = 62)	122.8 ± 101.7 (*n* = 124)
*Chol7*	1	non-HDL-C	2.093	66.95	rs6354736	279.5 ± 62.8 (*n* = 56)	257.8 ± 56.9 (*n* = 57)	251.2 ± 52.1 (*n* = 114)
*Nhdlq15*	2	non-HDL-C	2.56	23.86	mCV23209429	273.9 ± 56.1 (*n* = 55)	238.0 ± 47.0 (*n* = 53)	262.7 ± 59.0 (*n* = 120)
*Hdlq34*	5	non-HDL-C	2.106	19.4	rs3658401	244.5 ± 54.7 (*n* = 63)	276.0 ± 53.2 (*n* = 61)	259.3 ± 58.3 (*n* = 104)
*Pnhdlc1*	6	non-HDL-C	2.489	57.53	rs13478909	279.6 ± 51.3 (*n* = 51)	252.0 ± 65.2 (*n* = 57)	254.8 ± 53.5 (*n* = 120)
*Nhdlq1*	8	non-HDL-C	2.221	44.14	D8Mit50	275.0 ± 54.5 (*n* = 60)	242.4 ± 57.4 (*n* = 57)	262.9 ± 55.6 (*n* = 96)
*Nhdlq12*	12	non-HDL-C	2.73	39.41	rs6195664	278.6 ± 52.3 (*n* = 62)	243.8 ± 57.8 (*n* = 59)	257.4 ± 56.5 (*n* = 107)
***Nhdlq15***	**2**	non-HDL-W	**4.79**	31.8	rs13476507	954.1 ± 156.0 (*n* = 56)	806.9 ± 158.2 (*n* = 47)	915.6 ± 166.1 (*n* = 125)
*Nhdlq11*	9	non-HDL-W	2.136	32.37	rs3709825	958.4 ± 211.4 (*n* = 42)	856.8 ± 165.3 (*n* = 62)	906.6 ± 149.5 (*n* = 124)
*-*	11	non-HDL-W	2.332	1.99	rs4222040	927.3 ± 149.8 (*n* = 67)	849.0 ± 165.1 (*n* = 69)	917.0 ± 170.5 (*n* = 85)
***Nhdlq16***	**16**	non-HDL-W	**3.99**	46.66	rs3721202	820.2 ± 152.7 (*n* = 56)	931.9 ± 146.4 (*n* = 52)	928.4 ± 174.6 (*n* = 120)
*Tgq11*	2	Triglyceride-C	2.952	26.73	mCV23209429	123.7 ± 35.7 (*n* = 55)	101.9 ± 34.6 (*n* = 53)	107.3 ± 31.8 (*n* = 120)
*-*	5	Triglyceride-C	2.759	80.03	gnf05.120.578	110.2 ± 33.2 (*n* = 43)	119.3 ± 35.8 (*n* = 88)	101.6 ± 31.3 (*n* = 97)
*Trglyd*	1	Triglyceride-W	3.291	97.02	rs13476259	94.0 ± 28.6 (*n* = 59)	115.3 ± 33.0 (*n* = 62)	100.7 ± 30.8 (*n* = 106)

*Chr* chromosome, *LOD* logarithm of odds, *C* chow diet, *W* Western diet, *BB* homozygous BALB allele, *SS* homozygous SM allele, *SM* heterozygous allele

Data are mean ± SD. The units for these measurements are mg/dL for plasma glucose or lipid levels. The number in the brackets represents the number of progeny with a specific genotype at a peak marker. The significant QTLs and their LOD scores were highlighted in bold

### Fasting lipid levels

**Fig. 3:**
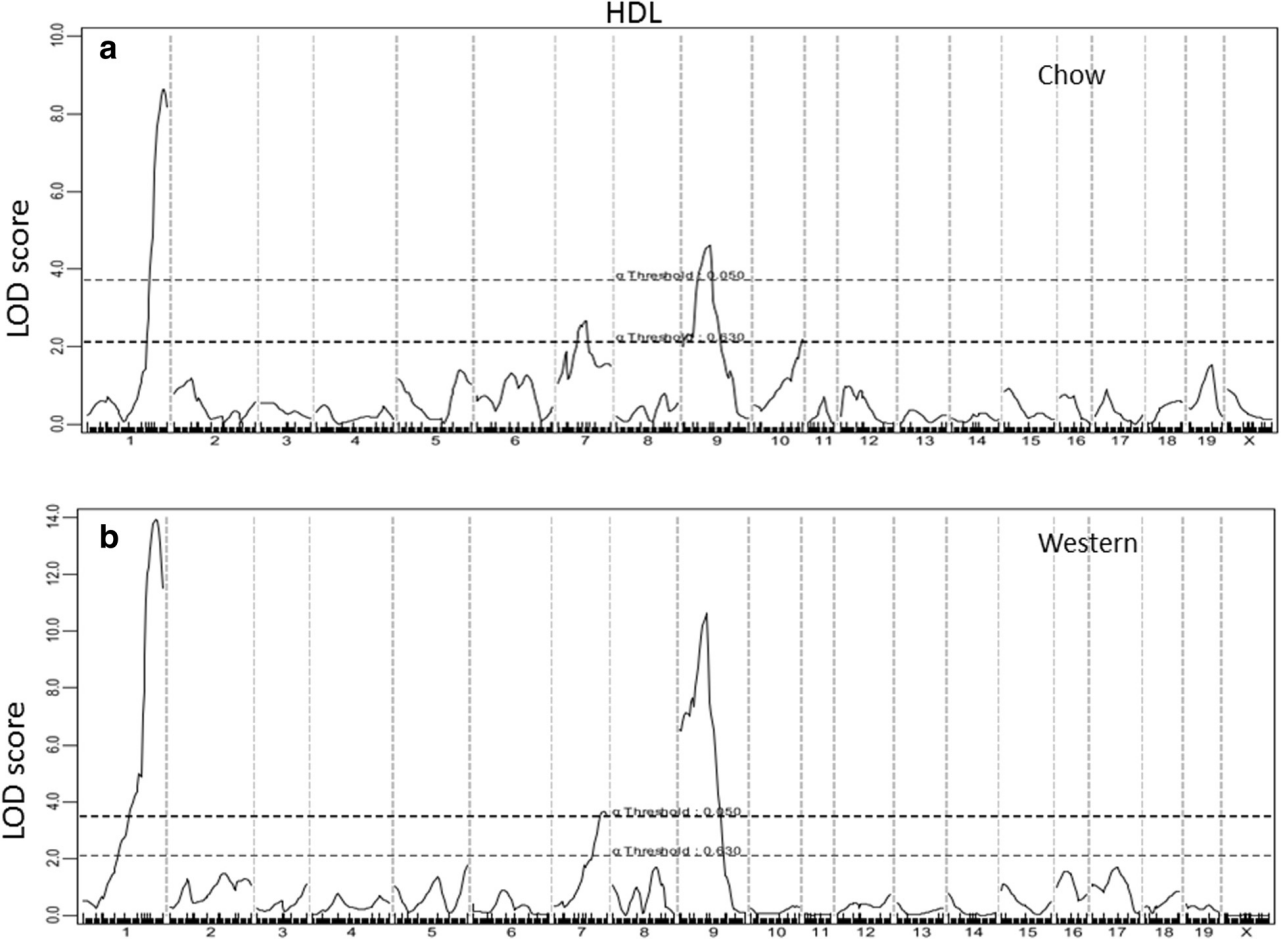
Genome-wide scans to search for loci influencing HDL cholesterol levels of female F_2_ mice when fed a chow (**a**) or Western diet (**b**). Three significant loci on chromosomes 1, 7, and 9 and one suggestive locus on chromosome 10 were detected to affect HDL cholesterol levels of mice

**Fig. 4:**
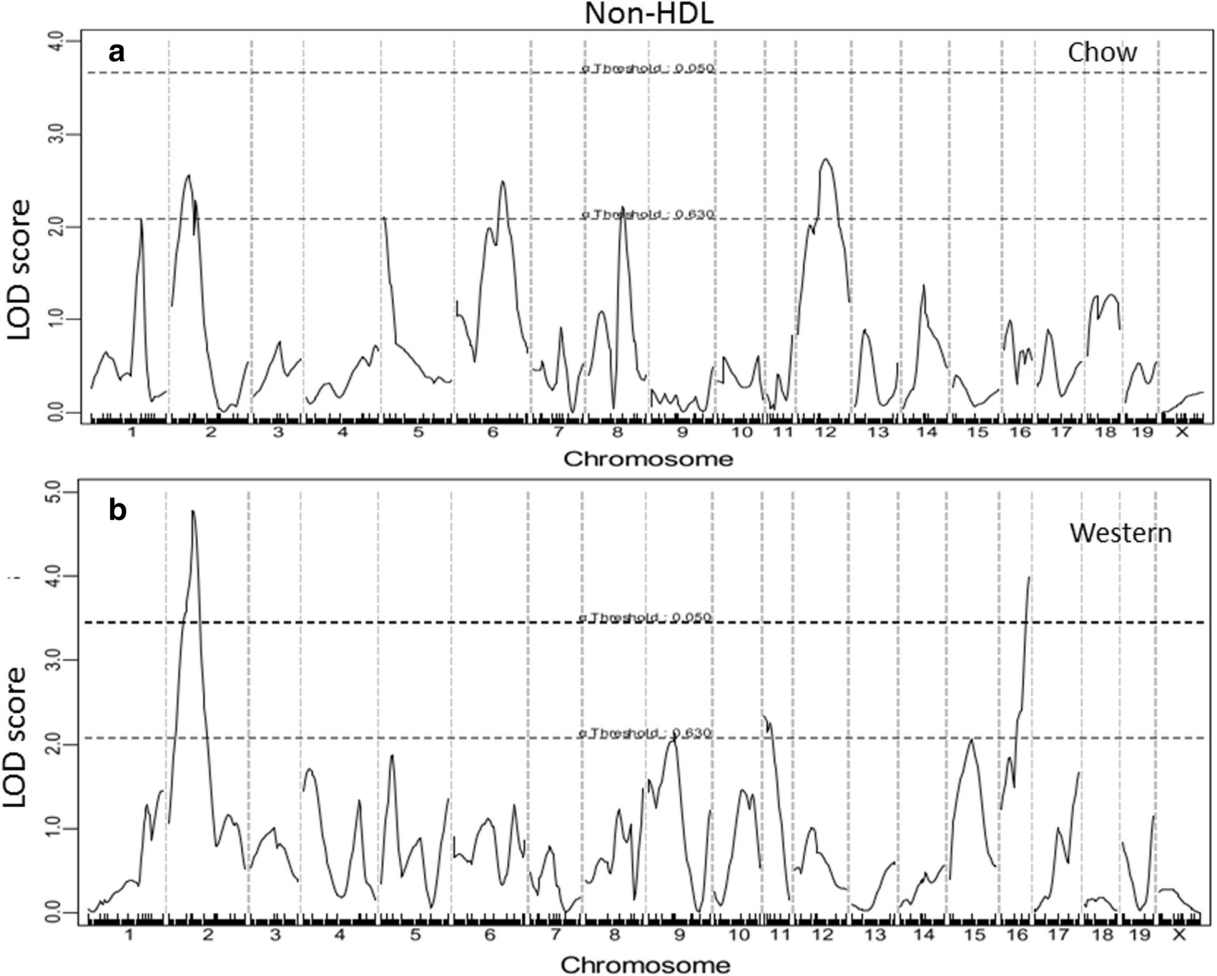
Genome-wide scans to search for loci influencing non-HDL cholesterol levels of female F_2_ mice fed a chow (**a**) or Western diet (**b**). Two significant loci on chromosomes 2 and 16 were identified to affect non-HDL cholesterol levels of mice fed the Western diet

**Fig. 5:**
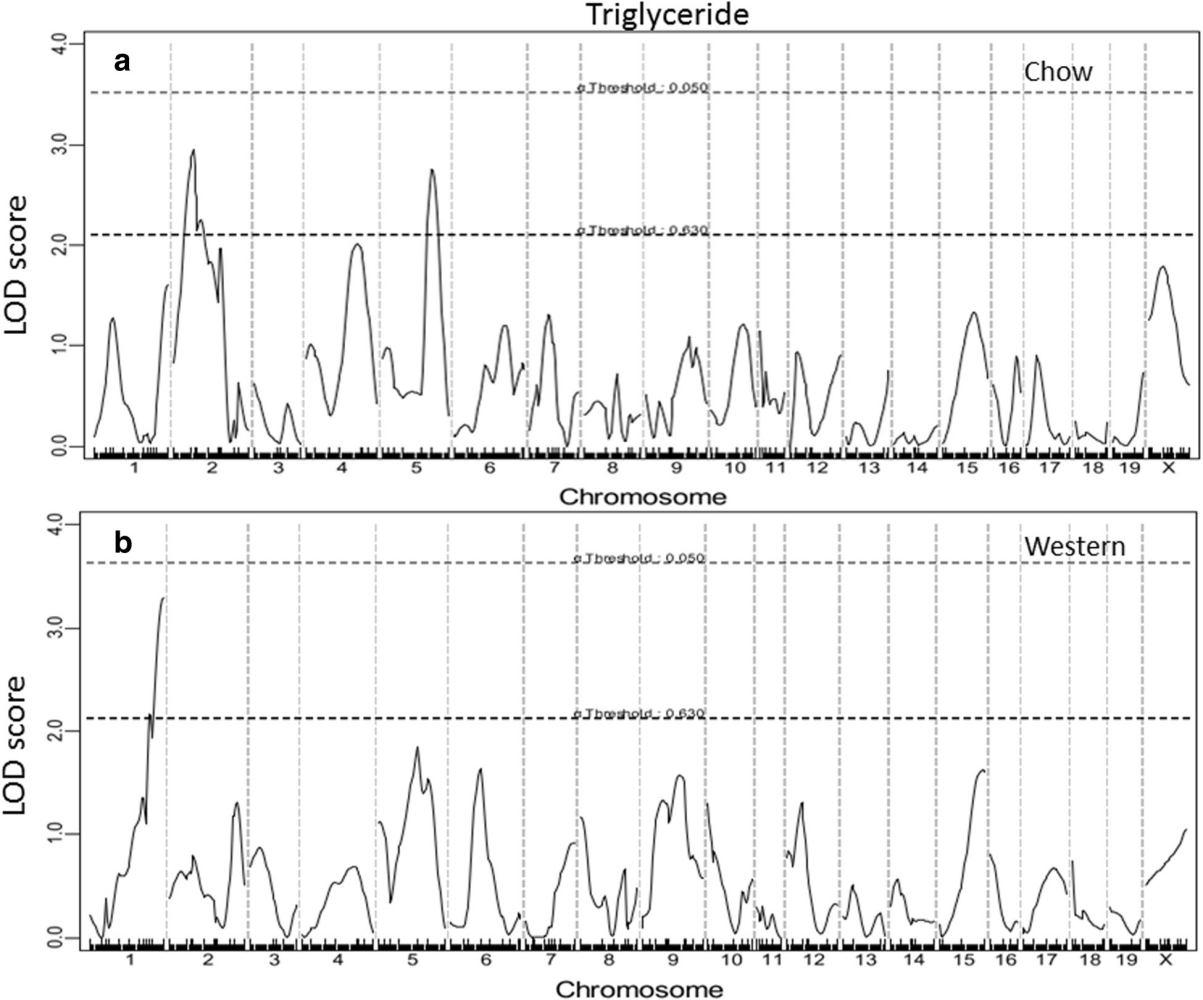
Genome-wide scans to search for loci influencing triglyceride levels of female F_2_ mice fed a chow (**a**) or Western diet (**b**). Three suggestive loci were identified for triglyceride levels

Genome-wide scans for main effect QTLs showed that HDL, non-HDL cholesterol, and triglyceride levels were each controlled by multiple QTLs (Figs. 3, 4 and 5; Table 1). For HDL, 3 significant QTLs, located on Chr1, Chr7 and Chr9, and 1 suggestive QTL on Chr10, were identified. All 3 significant QTLs for HDL were detected when mice were fed either chow or Western diet, while the suggestive QTL on Chr10 was found when mice were on the chow diet. The significant QTL on Chr1 replicated *Hdlq5,* which had been mapped in numerous crosses [25]. The Chr7 QTL replicated *Hdlcl1,* initially mapped in (PERA/EiJ x B6-*Ldlr*)) x B6-*Ldlr* backcross [26]. The Chr9 QTL replicated *Hdlq17*, previously mapped in B6 x 129S1/SvImJ F_2_ mice [27]. The suggestive QTL on Chr10 overlapped with *Hdlq26* mapped in a SM/J x NZB/BlNJ intercross [28]. For all 4 HDL QTLs, F_2_ mice homozygous for the SS allele had higher HDL levels than those homozygous for the BB allele (Table 2).

For non-HDL cholesterol levels, 6 suggestive QTLs were detected when F_2_ mice were fed the chow diet, and 2 significant and 2 suggestive QTLs were detected on the Western diet (Fig. 4). The 2 significant QTLs on Chr2 and Chr16 and the suggestive QTL on Chr11 were novel. The former 2 QTLs were named *Nhdlq15* and *Nhdlq16*, respectively. *Nhdlq15* peaked at 31.8 cM on Chr2 and affected non-HDL levels in a dominant mode from the BB allele while *Nhdlq16* peaked at 46.66 cM on Chr16 and affected non-HDL levels in a dominant mode from the SS allele. The rest replicated previously identified ones in other mouse crosses: The Chr1 QTL peaked at 66.95 cM, overlapping with *Chol7* mapped in an intercross of 129S1/SvImJ and CAST/Ei mice [29]. The Chr5 QTL overlapped with *Hdlq34* mapped in PERA/EiJ × I/LnJ and PERA/EiJ × DBA/2 J intercrosses [30]. The Chr6 QTL overlapped with *Pnhdlc1*, initially mapped in a B6 x CASA/Rk intercross and then replicated in B6 x C3H *Apoe*^−/−^ F_2_ mice [31, 32]. The Chr8 QTL replicated *Nhdlq1*, initially mapped in B6 x 129S1/SvImJ F_2_ mice [33]. The Chr9 QTL replicated *Nhdlq11*, initially mapped in B6 x C3H *Apoe*^−/−^ F_2_ mice [32]. The Chr12 QTL peaked at 44.14 cM, overlapping with *Nhdlq12* mapped in a B6 x C3H *Apoe*^−/−^ F_2_ intercross [32].

For triglyceride levels, 3 suggestive QTLs, located on Chr1, 2, and 5, respectively, were identified (Fig. 5). The Chr1 QTL peaked at 97 cM, 17 cM distal the *Apoa2* gene (80 cM). The Chr2 QTL replicated *Tgq11,* mapped in an intercross between DBA/1J and DBA/2J [34]. The Chr5 QTL was novel.

### Coincident QTLs for fasting glucose and lipids

**Fig. 6:**
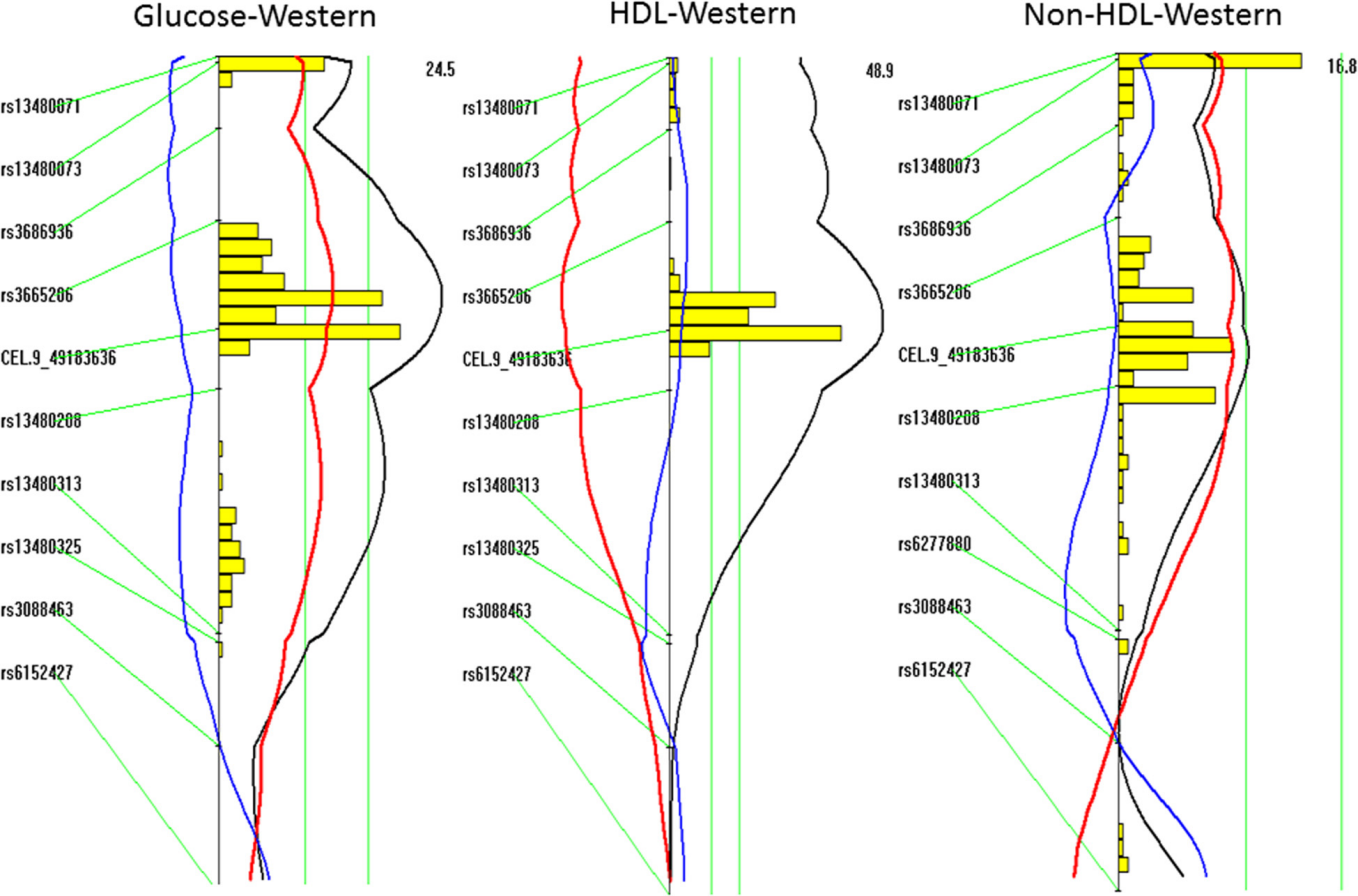
LOD score plots for fasting glucose, HDL, and non-HDL cholesterol of F_2_ mice fed the Western diet on chromosome 9. Plots were created with the interval mapping function of Map Manager QTX. The histogram in the plot estimates the confidence interval for a QTL. Two green vertical lines represent genome-wide significance thresholds for suggestive or significant linkage (*P* = 0.63 and *P* = 0.05, respectively). Black plots reflect the LOD score calculated at 1-cM intervals, the red plot represents the effect of the BALB allele, and the blue plot represents the effect of the SM allele. If BALB represents the high allele, then the red plot will be to the right of the graph; otherwise, it will be to the left

LOD score plots for Chr9 showed that the QTL for fasting glucose (*Bglu17*) coincided precisely with the QTLs for HDL (*Hdlq17*) and non-HDL (*Nhdlq11*) in the confidence interval (Fig. 6). F_2_ mice homozygous for the BB allele exhibited elevated levels of fasting glucose and non-HDL but decreased levels of HDL, compared to those homozygous for the SS allele (Table 2). These QTLs affected their respective trait values in an additive manner.

### Correlations between plasma glucose and lipid levels

**Fig. 7:**
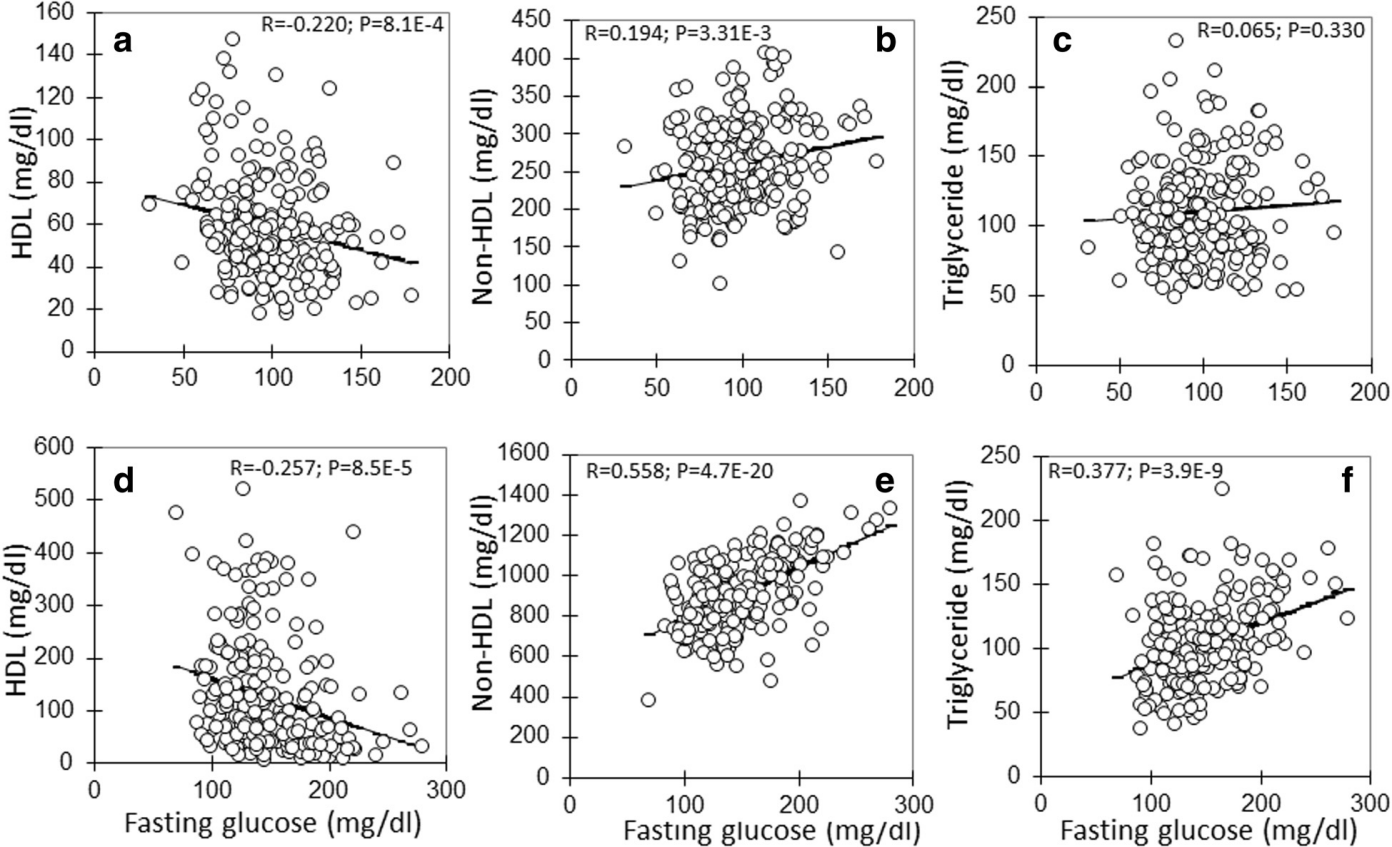
Correlations of fasting plasma glucose levels with plasma levels of HDL, non-HDL cholesterol and triglyceride in the F_2_ population fed a chow (top row: **a**, **b**, **c**) or Western diet (bottom row: **d**, **e**, **f**). Each point represents values of an individual F_2_ mouse. The correlation coefficient (*R*) and significance (*P*) are shown

The correlations of fasting glucose levels with plasma levels of HDL, non-HDL cholesterol, or triglyceride were analyzed with the F_2_ population (Fig. 7). A significant inverse correlation between fasting glucose and HDL cholesterol levels was observed when the mice were fed a chow (*R* = −0.220; *P* = 8.1E-4) or Western diet (*R* = −0.257; *P* = 8.5E-5). F_2_ mice with higher HDL cholesterol levels had lower fasting glucose levels. Conversely, significant positive correlations between fasting glucose and non-HDL cholesterol levels were observed when mice were fed either chow (*R* = 0.194; *P* = 3.31E-3) or Western diet (*R* = 0.558; *P* = 4.7E-20). F_2_ mice with higher non-HDL cholesterol levels also had higher fasting glucose levels, especially on the Western diet. A significant positive correlation between plasma levels of fasting glucose and triglyceride was observed when mice were fed the Western diet (*R* = 0.377; *P* = 3.9E-9) but not the chow diet (*R* = 0.065; *P* = 0.330).

### Prioritization of positional candidate genes for Chr9 coincident QTLs

*Bglu17* on Chr9 has been mapped in 3 separate intercrosses, including previously reported C57BLKS x DBA/2 [35] and B6-*Apoe*^−/−^ x BALB-*Apoe*^−/−^ crosses [21]. *Hdlq17* on Chr9 has been mapped in multiple crosses, including B6 x 129, B6 x CAST/EiJ, B6-*Apoe*^−/−^ x C3H-*Apoe*^−/−^, and B6-*Apoe*^−/−^ x BALB-*Apoe*^−/−^ crosses [24, 27, 31, 32, 36–38]. We conducted haplotype analyses using Sanger SNP database to prioritize positional candidate genes for both QTLs. Prioritized candidate genes for *Hdlq17* are shown in Additional file 1: Table S1, and candidate genes for *Bglu17* are shown in Additional file 2: Table S2. Most candidates for *Hdlq17* are also candidate genes for *Bglu17*. These candidates contain one or more non-synonymous SNPs in the coding regions or SNPs in the upstream regulatory region that are shared by the high allele strains but are different from the low allele strains at the QTL. All candidate genes were further examined for associations with relevant human diseases using the NIH GWAS database (http://www.genome.gov/GWAStudies/). *Sik3, Apoa1,* and *Apoc3* have been shown to be associated with variations in total, HDL, LDL-cholesterol or triglyceride levels [6, 7, 39], and *Cadm1* with obesity-related traits [40].

## Discussion

BALB and SM are two mouse strains that exhibit distinct differences in HDL, non-HDL cholesterol, and type 2 diabetes-related traits when deficient in *Apoe* [16]. BALB-*Apoe*^−/−^ mice have higher HDL, lower non-HDL cholesterol, and lower glucose levels than SM-*Apoe*^−/−^ mice when they are fed a Western diet. To identify the genetic factors responsible for these differences, we performed QTL analysis on a female cohort derived from an intercross between the two *Apoe*^−/−^ strains. We have identified four loci contributing to fasting glucose levels, four loci contributing to HDL cholesterol levels, nine loci for non-HDL cholesterol levels, and three loci for triglyceride levels. Moreover, we have observed genetic connections between dyslipidemia and type 2 diabetes in that the QTL for fasting glucose is colocalized with the QTLs for HDL and non-HDL cholesterol on chromosome 9 and these coincident QTLs share a large fraction of potential candidate genes.

We identified a significant QTL on chromosome 9, peaked at 26 cM, which affected fasting plasma glucose levels when mice were fed a chow or Western diet. We named it *Bglu17* to represent a novel locus regulating fasting glucose levels in the mouse. This locus is overlapping with a significant QTL (not named) for blood glucose levels on the intraperitoneal glucose tolerance test identified in a BKS-Cg-*Leprdb*^+/+*m*^ x DBA/2 intercross and a suggested QTL identified in a B6-*Apoe*^−/−^ x BALB-*Apoe*^−/−^ intercross [21, 35]. Interestingly, we found that *Bglu17* coincided precisely with *Hdlq17*, a QTL for HDL cholesterol levels, and *Nhdlq11*, a QTL for non-HDL cholesterol levels. The colocalization of two or more QTLs for different traits suggests that these traits are controlled either by the same gene(s) or closely linked but different individual genes. *Hdlq17* has been mapped in multiple crosses derived from inbred mouse strains whose genomes have been resequenced by Sanger, including B6, 129, BALB, C3H/HeJ, and CAST/EiJ [24, 27, 31, 32, 36–38]. *Nhdlq11* was previously mapped in a NZB/BINJ x SM/JF2 cross and a B6-*Apoe*^−/−^ x C3H-*Apoe*^−/−^ intercross [32, 41]. To determine whether *Bglu17* and *Hdlq17* share the same underlying candidate genes, we performed haplotype analyses on those crosses that led to the identification of the QTLs. The number of shared genetic variants between *Bglu17* and *Hdlq17* was surprisingly high*.* Of them, *Sik3*, *Apoa1*, and *Apoc3* are located precisely underneath the linkage peak of *Bglu17* and *Hdlq17*, and they are also functional candidate genes of *Hdlq17*. Indeed, recent GWAS studies have associated these three genes with dyslipidemia or variations in HDL, LDL cholesterol, and triglyceride levels [6, 39, 42]. The finding in this study strongly suggests that one or more of these “lipid genes” might be the causal gene(s) of *Bglu17*, contributing to variation in fasting glucose levels. Although it is unknown how they affect glucose homeostasis, one probable effect path is through the influence on plasma lipid levels, which then predispose variation in glucose-related traits. The current observation on the significant correlations of fasting glucose levels with HDL, non-HDL cholesterol, and triglyceride levels in this cross supports this speculation. Plasma lipid levels, especially non-HDL cholesterol, of the F_2_ mice were significantly elevated on the Western diet, so were the fasting glucose levels. When fed the Western diet, *Apoe*^−/−^ mice display a rapid rise in non-HDL cholesterol levels, often reaching their peak within a couple of weeks (unpublished data), whereas their blood glucose levels rise more slowly and gradually within 12 weeks [43, 44]. This difference in onset suggests a causal role for plasma lipids in the rise of blood glucose in the *Apoe*^−/−^ mouse model.

A significant reverse correlation was observed between plasma HDL cholesterol levels and fasting glucose levels in this cross on either chow or Western diet. This result is consistent with the findings of prospective human studies that low HDL levels can predict the future risk of developing T2D and low HDL levels are more prevalent in diabetic patients than in the normal population [45, 46]. HDL can increase insulin secretion from β-cells, improve insulin sensitivity of the target tissues, and accelerate glucose uptake by muscle via the AMP-activated protein kinase [47]. A significant correlation of non-HDL cholesterol levels with fasting glucose levels was also observed in this cross, and the correlation was extremely high when mice were fed the Western diet. Emerging human studies have also revealed associations of non-HDL cholesterol and ApoB with fasting glucose levels and incident type 2 diabetes [48–50]. We previously observed that the elevation of non-HDL cholesterol levels in *Apoe*^−/−^ mice during the consumption of a Western diet induces a chronic, low-grade inflammation state characterized by rises in circulating cytokines and infiltration of monocytes/macrophages in various organs or tissues [13, 17, 20, 43]. Inflammation in the islets impairs β-cell function [20]. LDL can also directly affect function and survival of β-cells [51]. In addition, high levels of LDL can induce insulin resistance due to its lipotoxicity and effect on endoplasmic reticulum stress [1].

Plasma triglyceride levels were strongly correlated with fasting glucose levels in this cross on the Western diet, although no significant correlation was found when mice were fed the chow diet. Despite the strong correlation, no overlapping QTLs were observed for fasting glucose and triglyceride. The reason for the discrepancy between non-HDL cholesterol and triglyceride in terms of the presence or absence of colocalized QTLs is unclear.

A suggestive QTL for fasting glucose near the proximal end of chromosome 9 (2.37 cM) was detected in this cross, initially on the chow diet and then replicated on the Western diet. The LOD score plot for chromosome 9 has shown 2 distinct peaks, one with a suggestive LOD score at the proximal end and one with a significant LOD score at a more distal region, suggesting the existence of two loci for fasting glucose on the chromosome. The bootstrap test, a statistical method for defining the confidence interval of QTLs using simulation [52], also indicated the existence of two QTLs for the trait on chromosome 9. We named the proximal one *Bglu16* to represent a new QTL for fasting glucose in the mouse. Naming a suggestive locus is considered appropriate if it is repeatedly observed [53].

Two suggestive QTLs for fasting glucose on chromosome 5 were identified when mice were fed the Western diet. The proximal one replicated *Bglu13*, recently mapped in the B6-Apoe^−/−^ x BALB-Apoe^−/−^ cross [21]. One probable candidate gene for this QTL is *Hnf1a*, which encodes hepatocyte nuclear factor 1α. In humans, *Hnf1a* mutations are the most common cause of maturity-onset diabetes of the young (MODY) [54]. The suggestive QTL in the distal region was novel.

Most of the QTLs identified for plasma lipids confirm those identified in previous studies, whereas two QTLs for non-HDL are new and named *Nhdlq15* and *Nhdlq16*, respectively. The QTLs on distal chromosome 1 for HDL and triglyceride has been mapped in a number of mouse crosses, and *Apoa2* has been identified as the underlying causal gene [55]. However, the QTL (~90 cM) mapped in this study showed that it was more distal to the *Apoa2* gene (80 cM), thus suggesting a different underlying causal gene.

## Conclusion

We have identified multiple QTLs contributing to dyslipidemia and hyperglycemia in a segregating F_2_ population. The finding on the colocalization of QTLs for fasting glucose, HDL and non-HDL cholesterol levels and the sharing of probable candidate genes has demonstrated genetic connections between dyslipidemia and type 2 diabetes. The close correlations of fasting glucose with HDL, non-HDL cholesterol, and triglyceride support the hypothesis that dyslipidemia plays a causal role in the development of type 2 diabetes [1]. The haplotype analysis has prioritized candidates for either chromosome 9 QTL down to a handful of genes. Nevertheless, functional studies need to be performed to prove causality.

## Availability of supporting data

Data are accessible through this link: https://mynotebook.labarchives.com/doi/MTc2Mzk0LjR8MTM1Njg4LzEzNTY4OC9Ob3RlYm9vay80MTAyOTgxMTQ4fDQ0Nzc3MC40/10.6070/H4D50K0X.

## Additional files

Additional file 1: Table S1. Haplotype analysis to prioritize candidate genes for HDL QTL *Hdlq17* on chromosome 9. Sanger SNP database (http://www.sanger.ac.uk/sanger/Mouse_SnpViewer/rel-1410) was used to prioritize candidate genes for this locus, which were mapped in 3 separate crosses derived from different inbred strains. A high allele strain is the one that displays a larger allelic effect on HDL level at the locus and a low allele strain is the one showing a smaller allelic effect on HDL at the locus. (PDF 86 kb) Additional file 2: Table S2. Haplotype for glucose QTL *Bglu17* on chromosome 9. Analysis was performed in the same way as for *Hdlq17* illustrated above. (PDF 82 kb)

## Abbreviations

QTL: Quantitative trait locus (QTL) analysis
LOD: Logarithm of odds ratio
T2D: Type 2 diabetes
LDL: Low-density lipoprotein
HDL: High-density lipoprotein
GWAS: Genome-wide association studies
Apoe: Apolipoprotein E
