# Supplementary material for: Genetic linkage of hyperglycemia and dyslipidemia in an intercross between BALB/cJ and SM/J Apoe-deficient mouse strains
Source: BMC Genet. 2015 Nov 10;16:133. doi: 10.1186/s12863-015-0292-y (PMC4641414; doi:10.1186/s12863-015-0292-y)
Supplement: Additional file 1: Table S1. — Haplotype analysis to prioritize candidate genes for HDL QTL Hdlq17 on chromosome 9. Sanger SNP database (http://www.sanger.ac.uk/sanger/Mouse_SnpViewer/rel-1410) was used to prioritize candidate genes for this locus, which were mapped in 3 separate crosses derived from different inbred strains. A high allele strain is the one that displays a larger allelic effect on HDL level at the locus and a low allele strain is the one showing a smaller allelic effect on HDL at the locus. (PDF 86 kb) [file 12863_2015_292_MOESM1_ESM.pdf]

Supplementary Table 1. Haplotype analysis for HDL QTL Hdlq17 on chromosome 9.

				Low allele		High allele			Amino acid	
**Chr**	**Position**	**Gene**	**dbSNP**	**C57Bl/6**	**BALB_cJ**	**129**	**CAST_EiJ**	**Consequence**	**substitution**	**GWAS**
9	42944353	Grik4	rs2618949	A	-	G	G	5_prime_utr_variant		
9	44101786	Mfrp	rs3259532	A	-	G	G	5_prime_utr_variant		
9	44101954	Mfrp	rs3259533	G	-	A	A	splice_region_variant		
9	44134532	Mcam	rs2511342	A	-	g	G	5_prime_utr_variant		
9	44233939	Cbl	rs2147625	C	-	g	G	5_prime_utr_variant		
9	44246854	Ccdc153	rs3061495	G	-	A	A	missense_variant	Cn RQ:193	
9	44248645	Pdzd3	rs3083281	A	-	G	G	missense_variant	Cn CR:393	
9	44264018	Nlrx1	rs3052760	A	-	C	C	missense_variant	**Cn SA:154**	
9	44268349	Nlrx1	rs5200534	G	-	A	A	5_prime_utr_variant		
9	44268447	Nlrx1	rs5184594	G	-	A	A	5_prime_utr_variant		
9	44268466	Nlrx1	rs4590532	A	-	C	C	5_prime_utr_variant		
9	44268467	Nlrx1	rs4854267	A	-	C	C	5_prime_utr_variant		
9	44268545	Nlrx1	rs4821534	T	-	C	C	5_prime_utr_variant		
9	44274513	Abcg4	rs3018998	T	-	A*	A*	splice_region_variant 3_prime_utr_variant		
9	44279001	Abcg4	rs2294885	G	-	C	C	missense_variant	Cn LV:347	
9	44281797	Abcg4	rs4594317	A	-	T	T	splice_region_variant		
9	44288250	Abcg4	rs2445065	A	-	G	G	5_prime_utr_variant		
9	44288343	Abcg4	rs2446606	C	-	G	G	5_prime_utr_variant		
9	44326936	Dpagt1	rs3018032	T	-	A	A	5_prime_utr_variant		
9	44332931	Dpagt1	rs3260894	T	-	C	C	missense_variant	**Cn IT:376**	
9	44334761	H2afx	rs1346379	T	-	C	C	5_prime_utr_variant		
9	44342260	Hmbs	rs2362819	G	-	A	A	5_prime_utr_variant		
9	44348897	Vps11	rs2974908	T	-	C	C	splice_region_variant		
9	44388141	Hyou1	rs2968712	G	-	A*	A*	missense_variant 3_pr	Cn DN:710	
9	44388270	Hyou1	rs2993490	T	-	C	C	splice_region_variant		
9	44407305	Trappc4	rs3260958	T	-	C	G	5_prime_utr_variant		
9	44413146	Ccdc84	rs3260878	G	-	A	A	missense_variant	Cn AV:190	
9	44435266	Foxr1	rs3678819	A	-	G	G	missense_variant	Cn YH:195	
9	44479414	C030014I2	rs4830044	G	-	C	C	splice_region_variant		
9	44556356	Cxcr5	rs3053446	A	-	T	T	5_prime_utr_variant		
9	44607365	Ddx6	rs3017598	G	-	A	A	5_prime_utr_variant		
9	44607516	Ddx6	rs3040312	C	-	A	A	missense_variant	Cn LM:42	Rheumatoi
9	44681315	Treh	rs3262009	A	-	G	G	missense_variant	Cn QR:71	Serum met
9	44681344	Treh	rs3262009	A	-	G	G	missense_variant	**Cn SG:81**	
9	44684660	Treh	rs3038329	T	-	A*	A*	missense_variant 3_pr	Cn ST:381	
9	44698113	Phldb1	rs3262109	A	-	G	G	missense_variant	**Cn SP:1082**	**Total chole**
9	44711884	Phldb1	rs3262204	A	-	G	G	5_prime_utr_variant		
9	44716606	Phldb1	rs3009097	A	-	C	C	missense_variant	**Cn SA:181**	
9	44718309	Phldb1	rs3364125	A	-	G	C	missense_variant	Cn VG:149	
9	44759955	Arcn1	rs3262327	T	-	C	C	splice_region_variant		
9	44817773	Mll1	rs3262891	T	-	C	C	missense_variant	Cn ND:3581	
9	44845631	Mll1	rs3262980	A	-	G	G	splice_region_variant		
9	44946178	Ube4a	rs2528500	A	-	G	G	splice_region_variant		
9	44981860	Cd3d	rs2992758	A	-	C	C	5_prime_utr_variant		
9	44985159	Cd3d	rs3018021	T	-	A	A	missense_variant	**Cn VE:82**	
9	44985161	Cd3d	rs3009136	G	-	A	A	missense_variant	Cn VM:83	
9	44985167	Cd3d	rs2960395	T	-	A	A	missense_variant	Cn ST:85	
9	44986343	Cd3d	rs3362378	A	-	G	G	stop_retained_variant		
9	45042723	Mpzl2	rs4855887	A	-	G	G	5_prime_utr_variant		
9	45062044	Mpzl3	rs3371231	C	-	T	T	missense_variant	Cn AV:27	
9	45062214	Mpzl3	rs2999555	G	-	A*	A*	missense_variant splic	Cn VM:84	
9	45079187	Amica1	rs3013676	T	-	C	C	5_prime_utr_variant		
9	45082679	Amica1	rs2505079	T	-	A	A	5_prime_utr_variant		
9	45093746	Amica1	rs4792711	T	-	G*	G*	missense_variant splic	Cn DE:145	
9	45093848	Amica1	rs4610910	C	-	G	G	missense_variant	Cn SR:179	
9	45103035	Amica1	rs3002621	C	-	T	T	splice_region_variant		
9	45256191	Il10ra	rs2152899	C	-	T*	T*	missense_variant 3_pr	**Cn GE:356**	Obesity-rel
9	45265618	Il10ra	rs4948494	C	-	G*	G*	missense_variant 3_pr	Cn MI:138	
9	45265619	Il10ra	rs1077590	A	-	G*	G*	missense_variant 3_pr	**Cn MT:138**	
9	45266550	Il10ra	rs2963971	T	-	C	C	missense_variant	Cn HR:94	
9	45370313	Fxyd6	rs3366705	A	-	G	G	5_prime_utr_variant		
9	45370332	Fxyd6	rs4674062	T	-	C	C	5_prime_utr_variant		
9	45370353	Fxyd6	rs2295461	G	-	C	C	5_prime_utr_variant		
9	45408152	Fxyd2	rs4946308	G	-	C	C	splice_region_variant		
9	45710207	Dscaml1	rs2595496	T	-	C	C	splice_region_variant		
9	45712228	Dscaml1	rs3264645	C	-	T	T	splice_region_variant		
9	45773838	Cep164	rs3047898	T	-	C	C	missense_variant	Cn MV:1035	
9	45775315	Cep164	rs3697381	T	-	C	C	missense_variant	Cn HR:867	
9	45775734	Cep164	rs4819440	T	-	C	C	missense_variant	Cn IM:830	
9	45775750	Cep164	rs4988088	A	-	G	G	splice_region_variant		
9	45776930	Cep164	rs3264097	G	-	A	A	splice_region_variant		
9	45779147	Cep164	rs3024264	T	-	C	C	missense_variant	Cn QR:664	
9	45779404	Cep164	rs2513472	T	-	A	A	missense_variant	**Cn QL:632**	
9	45779796	Cep164	rs2267032	T	-	C	C	missense_variant	Cn KE:528	
9	45794135	Cep164	rs5249480	T	-	C	C	missense_variant	Cn KE:355	
9	45871632	Rnf214	rs3265199	T	-	G*	G*	splice_region_variant 3_prime_utr_variant		
9	45890796	Rnf214	rs2606362	G	-	A	A	splice_region_variant		
9	45906850	Pcsk7	rs1679348	T	-	C	C	5_prime_utr_variant		Cardiovasc
9	45935898	Tagln	rs4782762	C	-	T	T	5_prime_utr_variant		
9	45947062	Sidt2	rs2532832	T	-	C	C	splice_region_variant		
9	45947866	Sidt2	rs3018098	A	-	C	C	missense_variant	Cn LV:329	
9	46123341	Sik3	rs4992923	G	-	A	A	splice_region_variant		**HDL choles**
9	46198304	Sik3	rs3267147	G	-	C	C	splice_region_variant		
9	46209671	Sik3	rs3267459	A	-	G	G	splice_region_variant		
9	46234722	Apoc3	rs2988967	C	-	T*	T*	missense_variant 5_prime_utr_variant		**Heart disea**
9	46235183	Apoc3	rs2988177	A	-	C	C	missense_variant	**Cn SF:258**	
9	50528620	Pts	rs3035302	T	-	C	C	missense_variant	Cn DG:6	
9	50536299	Bco2	rs6386942	C	-	G	G	missense_variant	Cn AP:375	Inflammati
9	50550728	Bco2	rs2216841	A	-	G	G	5_prime_utr_variant		
9	50555141	Bco2	rs3274301	T	-	C	C	5_prime_utr_variant		
9	50581487	Il18	rs2402869	T	-	C	C	splice_region_variant		
9	50603903	Timm8b	rs3274490	A	-	G	G	5_prime_utr_variant		
9	50617554	Pih1d2	rs2586050	G	-	C	C	5_prime_utr_variant		
9	50618100	Pih1d2	rs3722296	A	-	G	G	5_prime_utr_variant		
9	50624966	Pih1d2	rs3274774	C	-	T*	T*	missense_variant 3_pr	Cn AV:307	
9	50646104	Dlat	rs4734454	C	-	T	T	splice_donor_variant		
9	50693690	Dixdc1	rs3275078	A	-	G	G	splice_region_variant		
9	50695466	Dixdc1	rs2998118	A	-	G	G	splice_region_variant		
9	50778963	Alg9	rs3031240	C	-	T	T	splice_region_variant		
9	50895683	Sik2	rs2988169	T	-	C	C	missense_variant	Cn QR:809	
9	50896707	Sik2	rs2225495	C	-	T	T	missense_variant	Cn SN:695	
9	51008921	Sik2	rs2629431	T	-	g	G	5_prime_utr_variant		
9	51063246	Layn	rs2155663	A	-	T	T	missense_variant	**Cn WR:198**	Obesity-rel
9	51116004	Btg4	rs3276411	T	-	C	C	5_prime_utr_variant		
9	53456706	Atm	rs2997257	C	-	T	T	splice_region_variant		Rheumatoi
9	53458931	Atm	rs4845546	T	-	G	G	missense_variant	Cn IL:2294	

Chr: chromosome; CN (Coding nonsynonymous SNP) followed by 1-letter abbreviation of substituted amino acid and its position in protein product. Substitutions with likely functional significance are denoted in bold. * Multiple consequences
