# Supplementary material for: Genetic linkage of hyperglycemia and dyslipidemia in an intercross between BALB/cJ and SM/J Apoe-deficient mouse strains
Source: BMC Genet. 2015 Nov 10;16:133. doi: 10.1186/s12863-015-0292-y (PMC4641414; doi:10.1186/s12863-015-0292-y)
Supplement: Additional file 2: Table S2. — Haplotype for glucose QTL Bglu17 on chromosome 9. Analysis was performed in the same way as for Hdlq17 illustrated above. (PDF 82 kb) [file 12863_2015_292_MOESM2_ESM.pdf]

Supplementary Table 2. Haplotype analysis for glucose QTL Bglu17 on Chr9.

				Low allele	High				Amino acid	
**Chr**	**Position**	**Gene**	**dbSNP**	**C57BL/6**	**BALB_cJ**	**C3H_HeJ**	**DBA_2J**	**Consequence**	**substitution**	**GWAS**
9	41984438	Sorl1	rs48859022	C	T	T	T	missense_variant	Cn RK:1807	Cardiovascular disease
9	41996288	Sorl1	rs45639841	A	G	G	G	missense_variant	Cn FL:1468	
9	42000339	Sorl1	rs36294187	T	C	C	C	missense_variant	Cn HR:1425	
9	42255371	Sc5d	rs36310583	C	T	T	T	missense_variant	**Cn GS:291**	
9	42264151	Sc5d	rs36768880	C	T	T	T	5_prime_utr_variant	-	
9	42330251	Tecta	rs33756006	T	C	C	C	missense_variant	Cn IV:2148	
9	42359326	Tecta	rs50440510	C	T	T	T	missense_variant	Cn VI:1229	
9	42367117	Tecta	rs30224599	G	T	T	T	missense_variant	**Cn PT:1032**	
9	42971205	Arhgef12	rs29892489	C	T	T	T	missense_variant	**Cn AT:1448**	
9	43310799	Trim29	rs33771075	T	C	C	C	5_prime_utr_variant	-	Stroke
9	43803750	Pvrl1	rs30034385	G	A	A	A	missense_variant	**Cn GS:428**	
9	44556292	Cxcr5	rs30926959	C	T	T	T	5_prime_utr_variant	-	Rheumatoid
9	44715942	Phldb1	rs29984860	C	T	T	T	missense_variant	Cn RH:402	**Cholesterol,**
9	44798980	Tmem25	rs33639727	T	C	C	C	5_prime_utr_variant	-	
9	44822144	Mll1	rs30076434	G	A	A	A	missense_variant	**Cn SL:2289**	
9	44848089	Mll1	rs30136208	A	G	G	G	missense_variant	Cn LP:821	
9	44848133	Mll1	rs30374209	T	A	A	A	missense_variant	Cn ED:806	
9	44944880	Ube4a	rs29586362	G	A	A	A	missense_variant	**Cn TM:499**	Fat distribution
9	45009588	Cd3e	rs49634626	C	A	A	A	5_prime_utr_variant	-	
9	45328525	Tmprss13	rs52431095	A	G	G	G	missense_variant	**Cn TA:44**	
9	45328610	Tmprss13	rs51091355	T	C	C	C	missense_variant	Cn LP:72	
9	45749732	Dscaml1	rs32648240	G	C	C	C	missense_variant	**Cn KN:1727**	Triglycerides
9	46212174	Sik3	rs32676148	A	C	C	C	missense_variant	Cn QH:1090	**HDL cholesterol, Triglyc**
9	46221115	Sik3	rs29644396	G	A	A	A	missense_variant	Cn SN:1335	
9	46230352	Apoa1	rs13462139	C	A	A	A	missense_variant	Cn QK:249	**Lipid traits, Triglyceride**
9	46230356	Apoa1	rs13462138	T	C	C	C	missense_variant	Cn VA:250	
9	46233317	Apoc3	rs32674712	G	A	A	A	missense_variant	**Cn SF:69**	**Lipid traits, Triglyceride**
9	47530231	Cadm1	rs46400319	T	C	C	C	5_prime_utr_variant	-	Obesity-related traits
9	48319917	Nxpe2	rs29976443	A	T	T	T	missense_variant	**Cn FY:384**	
9	48323052	Nxpe2	rs51271082	G	A	A	A	missense_variant	Cn PL:305	
9	48326428	Nxpe2	rs30183699	T	C	C	C	missense_variant	Cn IV:176	
9	48326470	Nxpe2	rs30361821	C	T	T	T	missense_variant	**Cn AT:162**	
9	48326482	Nxpe2	rs30183537	G	T	T	T	missense_variant	Cn LM:158	
9	48326493	Nxpe2	rs48981003	G	A	A	A	missense_variant	**Cn SF:154**	
9	48326495	Nxpe2	rs29689918	C	T	T	T	missense_variant	MI:153	
9	48339554	Nxpe2	rs46739467	G	A	A	A	missense_variant	Cn AV:31	
9	48339587	Nxpe2	rs48286201	T	C	C	C	missense_variant	Cn QR:20	
9	48353407	Nxpe2	rs47271733	C	T	T	T	5_prime_utr_variant	-	
9	48362047	Nxpe4	rs49529643	T	A	A	A	5_prime_utr_variant	-	
9	48389479	Nxpe4	rs46252680	G	A	A	A	5_prime_utr_variant	-	Ulcerative colitis
9	48389492	Nxpe4	rs49189452	A	T	T	T	5_prime_utr_variant	-	
9	48393023	Nxpe4	rs29927469	A	C	C	C	missense_variant	Cn ML:137	
9	48393039	Nxpe4	rs30136314	T	C	C	C	missense_variant	**Cn FS:142**	
9	48393131	Nxpe4	rs51400475	A	C	C	C	missense_variant	**Cn TP:173**	
9	48396492	Nxpe4	rs33702650	A	G	G	G	missense_variant	**Cn TA:299**	
9	48396531	Nxpe4	rs33732329	T	G	G	G	missense_variant	**Cn SA:312**	
9	48495360	Gm5617	rs51011504	G	C	C	C	5_prime_utr_variant	-	
9	48605019	Nnmt	rs8265570	G	A	A	A	5_prime_utr_variant	-	
9	48833071	Zbtb16	rs46982661	G	A	A	A	5_prime_utr_variant	-	
9	48833073	Zbtb16	rs49220368	T	G	G	G	5_prime_utr_variant	-	
9	48833079	Zbtb16	rs51719249	T	C	C	C	5_prime_utr_variant	-	
9	48835813	Zbtb16	rs3698611	A	G	G	G	5_prime_utr_variant	-	
9	48835833	Zbtb16	rs3698649	T	C	C	C	5_prime_utr_variant	-	
9	49064117	Zw10	rs13473788	A	G	G	G	missense_variant	**Cn TA:281**	
9	49421786	Ankk1	rs13480182	G	A	A	A	missense_variant	Cn LF:133	
9	49426936	Ankk1	rs29745186	G	T	T	T	missense_variant	**Cn AE:19**	
9	49427000	Ankk1	rs29991419	C	T	T	T	5_prime_utr_variant	-	
9	49440188	Ttc12	rs30669507	C	T	T	T	missense_variant	**Cn AT:670**	
9	50657959	Dlat	rs30191358	A	T	T	T	missense_variant	Cn DE:181	
9	50695485	Dixdc1	rs32752771	A	G	G	G	missense_variant	**Cn IT:278**	
9	50727900	Dixdc1	rs32748587	T	C	C	C	5_prime_utr_variant	-	
9	50727972	Dixdc1	rs32748589	C	T	T	T	5_prime_utr_variant	-	
9	50737727	Dixdc1	rs32747991	C	T	T	T	5_prime_utr_variant	-	
9	50739383	Dixdc1	rs32749419	T	C	C	C	5_prime_utr_variant	-	
9	50739404	Dixdc1	rs32749421	A	T	T	T	5_prime_utr_variant	-	
9	50739496	Dixdc1	rs32749423	A	C	C	C	5_prime_utr_variant	-	
9	50752960	Cryab	rs30286802	G	A	A	A	5_prime_utr_variant	-	
9	50768613	Fdxacb1	rs29939623	G	A	A	A	5_prime_utr_variant	-	
9	50770742	Fdxacb1	rs32754713	G	A	A	A	missense_variant	Cn RQ:215	
9	50775213	Alg9	rs48553853	A	G	G	G	5_prime_utr_variant	-	
9	50775337	Alg9	rs47034110	C	T	T	T	5_prime_utr_variant	-	
9	50775343	Alg9	rs29640131	T	C	C	C	5_prime_utr_variant	-	
9	51116640	Btg4	rs13461391	A	G	G	G	missense_variant	Cn IV:37	
9	51117988	Btg4	rs13461390	A	G	G	G	missense_variant	Cn IV:158	
9	52144261	Zc3h12c	rs51475643	C	T	T	T	missense_variant	Cn VI:83	
9	54286770	Gldn	rs29736523	T	C	C	C	missense_variant	**Cn SP:83**	

Chr: chromosome; CN (Coding nonsynonymous SNP) followed by 1-letter abbreviation of substituted amino acid and its position in protein product.
Substitutions with likely functional significance are denoted in bold.
